# Impact of insulin therapy on the mortality of acute heart failure patients with diabetes mellitus

**DOI:** 10.1186/s12933-021-01370-y

**Published:** 2021-09-08

**Authors:** Se Yong Jang, Jieun Jang, Dong Heon Yang, Hyun-Jai Cho, Soo Lim, Eun-Seok Jeon, Sang Eun Lee, Jae-Joong Kim, Seok-Min Kang, Sang Hong Baek, Myeong-Chan Cho, Dong-Ju Choi, Byung-Su Yoo, Kye Hun Kim, Sue K. Park, Hae-Young Lee

**Affiliations:** 1 Department of Internal Medicine, School of Medicine, Kyungpook National University, Daegu, Republic of Korea; 2 Department of Preventive Medicine, Korea University College of Medicine, Seoul, Republic of Korea; 3 Department of Internal Medicine, Seoul National University Hospital, Seoul, Republic of Korea; 4 Department of Internal Medicine, Seoul National University Bundang Hospital, Seongnam, Republic of Korea; 5 Department of Internal Medicine, Sungkyunkwan University College of Medicine, Seoul, Republic of Korea; 6 Department of Internal Medicine, Asan Medical Center, University of Ulsan College of Medicine, Seoul, Republic of Korea; 7 Department of Internal Medicine, Yonsei University College of Medicine, Seoul, Republic of Korea; 8 Department of Internal Medicine, College of Medicine, The Catholic University of Korea, Seoul, Republic of Korea; 9 Department of Internal Medicine, Chungbuk National University College of Medicine, Cheongju, Republic of Korea; 10 Department of Internal Medicine, Yonsei University Wonju College of Medicine, Wonju, Republic of Korea; 11 Department of Internal Medicine, Heart Research Center of Chonnam National University, Gwangju, Republic of Korea; 12 Department of Preventive Medicine, Seoul National University College of Medicine, Seoul, South Korea; 13 Department of Integrated Major in Innovative Medical Science, Seoul National University Graduate School, Seoul, Republic of Korea; 14 Cancer Research Institute, Seoul National University Graduate School, Seoul, Republic of Korea; 15 Division of Cardiology, Department of Internal Medicine, Seoul National University College of Medicine, 101 Daehak‑ro, Jongno‑gu, Seoul, 03080 South Korea

**Keywords:** Insulin, Diabetes mellitus, Heart failure, Mortality

## Abstract

**Background:**

Patients with diabetes mellitus (DM) have a higher prevalence of heart failure (HF) than those without it. Approximately 40 % of HF patients have DM and they tend to have poorer outcomes than those without DM. This study evaluated the impact of insulin therapy on mortality among acute HF patients.

**Methods:**

A total of 1740 patients from the Korean Acute Heart Failure registry with DM were included in this study. The risk of all-cause mortality according to insulin therapy was assessed using the Cox proportional hazard models with inverse probability of treatment weighting to balance the clinical characteristics (pretreatment covariates) between the groups.

**Results:**

DM patients had been treated with either oral hypoglycemic agents (OHAs) alone (n = 620), insulin alone (n = 682), or insulin combined with OHAs (n = 438). The insulin alone group was associated with an increased mortality risk compared with the OHA alone group (HR = 1.41, 95 % CI 1.21–1.66]). Insulin therapy combined with OHAs also showed an increased mortality risk (HR = 1.29, 95 % CI 1.14–1.46) compared with the OHA alone group. Insulin therapy was consistently associated with increased mortality risk, regardless of the left ventricular ejection fraction (LVEF) or HF etiology. A significant increase in mortality was observed in patients with good glycemic control (HbA1c < 7.0 %) receiving insulin, whereas there was no significant association in patients with poor glycemic control (HbA1c ≥ 7.0%).

**Conclusions:**

Insulin therapy was found to be associated with increased mortality compared to OHAs. The insulin therapy was harmful especially in patients with low HbA1c levels which may suggest the necessity of specific management strategies and blood sugar targets when using insulin in patients with HF.

**Supplementary Information:**

The online version contains supplementary material available at 10.1186/s12933-021-01370-y.

## Introduction

The prevalence of diabetes mellitus (DM) has increased by 29% over the past decade, affecting 475 million people worldwide in 2017 [1]. It is also a common comorbid condition in heart failure (HF) patients, affecting approximately 26–43% [2–7]. Patients with both HF and DM have been shown to have a poorer outcome than those with HF alone [8, 9], stressing the need to establish an optimal treatment strategy in order to improve their prognosis.

Some hypoglycemic agents used in the management of DM have been shown to have adverse effects in HF patients. Thiazolidinedione, once a bestselling hypoglycemic agent, is no longer recommended due to its fluid retention adverse effect [10, 11]. Insulin has also been associated with sodium retention and weight gain, potentially exerting harmful effects. Moreover, frequent insulin-related hypoglycemic events can be dangerous in HF patients. However, HF patients have often been prescribed insulin due to failure of glycemic control with oral hypoglycemic agents (OHAs) and lifestyle modifications.

Although some observational data showed a worse prognosis related to insulin therapy, conflicting results exist regarding the effect of insulin therapy on HF patients [12–16]. However, no randomized clinical trials have been conducted. Therefore, there is a need for large-scale, long-term studies to evaluate the effect of insulin therapy on HF patients. This study aimed to investigate the impact of insulin therapy on HF patients with DM using data from the Korean Acute Heart Failure (KorAHF) registry.

## Methods

### Data collection and study design

The KorAHF registry is a multicenter prospective cohort study conducted in ten tertiary hospitals in the Republic of Korea. The registry enrolled 5625 patients with acute HF between March 2011 and February 2014 and has been followed up until December 2019 [7, 17]. The patients’ baseline demographic data, laboratory and echocardiographic data, medication history, and clinical events were collected using the web-based Clinical Research and Trial system from the Korea Disease Control and Prevention Agency. All study protocols were reviewed and approved by the Institutional Review Board of each participating hospital, and registered with ClinicalTrials.gov (NCT01389843).

Two study settings were implemented to assess the impact of insulin therapy on the mortality of HF patients with DM. The first setting compared the mortality risk between those treated with insulin alone and those treated with OHAs alone, while the other setting compared the mortality risk between those with and without insulin therapy, regardless of combined OHAs.

### Study subjects and outcome assessment

Among all KorAHF patients, 2433 with DM were identified. DM was defined through patient self-reported history, glycated hemoglobin (HbA1c) level ≥ 6.5%, or the presence of hypoglycemic agents at baseline. Patients with missing information regarding pretreatment covariates were excluded due to the inability to estimate the propensity score, which is the probability of undergoing insulin therapy. Among the 2005 eligible patients, only those with available data on their hypoglycemic agent prescription were included. Patients with DM were divided based on their hypoglycemic agent into OHAs alone (OHA-only group, n = 620), insulin alone (insulin-only group, n = 682), or a combination of insulin and OHAs (all-insulin group = 438) (Additional file 1: Figure S1). Combination of insulin and OHAs group were defined as those who took only insulin or insulin along with OHAs. Information on the usage and type of DM medication was collected at the time of registry enrollment, and the registration date was defined as the time of exposure. The types of OHAs used among patients in this study were sulfonylureas, metformin, thiazolidinedione, acarbose, and dipeptidyl peptidase-4 inhibitors (Additional file 1: Table S1). Also, the specific types of insulin treatment are retrospectively reviewed and listed in Additional file 1: Table S2.

Patient deaths were identified by linking the Statistics Korea database’s death data to the registry data (among the study patients from 2011 to 2018). All deaths after registration were included in all the analyses.

### Statistical analyses

The patients’ clinical characteristics, including demographics, physical and laboratory findings, HF risk factors, and management are presented as frequencies and percentages or means and standard deviations. Clinical characteristics between the OHA alone group and insulin alone group or combination of insulin and OHAs group were compared using the chi-squared test for categorical variables and Student’s *t*-test for continuous variables.

We applied the generalized boosted model to estimate the inverse probability treatment weight (IPTW), which can consider many pretreatment covariates and reflect the nonlinear and complex associations between pretreatment covariates and treatment. Pretreatment covariates included age, sex, body mass index (BMI), history of hypertension, ischemic heart disease, atrial fibrillation, chronic lung disease, chronic kidney disease, cerebrovascular disease, systolic blood pressure, diastolic blood pressure, heart rate, glucose level, B-type natriuretic peptide levels (BNP) ≥ 500 pg/mL, N-terminal pro-B-type natriuretic peptide levels (NT-proBNP) ≥ 1000 pg/mL, serum sodium, potassium, blood urea nitrogen, and creatinine levels, New York Heart Association (NYHA) Class III-IV, and left ventricular ejection fraction (LVEF). Management by angiotensin-converting enzyme inhibitors (ACEIs), angiotensin II receptor blockers (ARBs), beta-blockers (BB), mineralocorticoid receptor antagonists (MRAs), warfarin, diuretics, inotropes, and vasodilators were all considered. Using standardized mean differences, propensity score matching was performed to balance the clinical characteristics (pretreatment covariates) between the OHA alone group and insulin alone group or combination of insulin and OHAs group. Imbalanced covariates were considered as those showing a standardized mean difference > 0.1, with statistical significance (*p* < 0.05).

Association between insulin therapy and mortality risk was evaluated by calculating hazard ratios (HRs) and 95 % confidence intervals based on the weighted Cox proportional hazard regression with IPTW. Although IPTW was used to obtain a pseudo-population with balanced clinical characteristics between the OHA alone group and insulin alone group or combination of insulin and OHAs group, there were still several imbalanced characteristics, including age, history of hypertension, treatment with intravenous vasodilators, and inotropic drugs, ACEIs, and ARBs. The imbalanced covariates were double-adjusted in the weighted Cox proportional hazard regression model to ensure unbiased estimates in the model and to reduce residual confounding.

Based on the assumption that the mortality according to DM medication among HF patients with DM would differ according to age, sex, LVEF, HbA1c level, HF of ischemic etiology, and severity of HF, the mortality risk evaluation of insulin therapy compared to that of oral therapy was performed in a population stratified according to age (< 65 years and ≥ 65 years), sex, etiology of HF (non-ischemic and ischemic), LVEF (< 40 % and ≥ 40 %), HbA1c level (< 7.0 % and ≥ 7.0 %), NYHA class (I-II and III-IV), and natriuretic peptide level (BNP and NT-proBNP cutoffs of 500 pg/mL and 1000 pg/mL). The mortality risk associated with insulin therapy with the two most frequently used classes of OHAs (sulfonylurea and metformin) was also evaluated. Statistical analyses were performed with an alpha error of 5 % using the R statistical software (version 3.6.2) with the “twang” and “survival” packages.

## Results

### Baseline characteristics

A total of 1740 patients with HF and DM were included in this study. Among those patients, 0.9% were diagnosed with type 1 DM. When comparing patients in the insulin alone group with those in the OHA alone group, patients receiving insulin were significantly more likely to be younger and have a lower BMI. They also had significantly lower rates of hypertension, but higher rates of chronic kidney disease, and inotrope and vasodilator use during the index admission. Despite both groups having similar LVEF, patients receiving insulin were significantly more likely to have severe symptoms, apparent by the higher NYHA classification. The prescription rates of ACEI/ARBs, BBs, and MRAs were significantly lower in the insulin-only group (Table 1).

**Table 1:** Baseline characteristics according to diabetes therapy in original cohort and inverse probability of treatment weighted pseudo-cohort within the Korean Acute Heart Failure (KorAHF) registry

	Original cohort					Weighted psueco-cohort					
	OHA-only (N = 620)	Insulin-only (N = 682)		All insulin (N = 1120)		OHA-only (N = 426)	Insulin-only (N = 682)		OHA-only (N = 697)	All insulin (N = 1120)	
	Mean (SD)	Mean (SD)	ASD	Mean (SD)	ASD	Mean (SD)	Mean (SD)	ASD	Mean (SD)	Mean (SD)	ASD
Age	71.2 (11.1)	67.3 (14.1)	− 0.28^1^	68.5 (12.8)	− 0.21^1^	69.9 (11.2)	67.3 (14.1)	− 0.19^1^	70.1 (10.8)	68.5 (12.8)	− 0.12^1^
BMI (kg/m^2^)	24.1 (3.7)	23.4 (3.9)	− 0.18^1^	23.4 (3.8)	− 0.18^1^	23.6 (3.8)	23.4 (3.9)	− 0.03	23.4 (3.6)	23.4 (3.8)	0.01
SBP (mmHg)	134.4 (28.4)	130.0 (32.7)	− 0.14^1^	132.2 (32.9)	− 0.07	135.0 (32.0)	130.0 (32.7)	− 0.15	135.0 (32.0)	132.2 (32.9)	− 0.09
DBP (mmHg)	79.5 (17.7)	75.6 (19.6)	− 0.20^1^	76.5 (19.3)	− 0.16^1^	77.7 (18.8)	75.6 (19.6)	− 0.11	77.7 (18.8)	76.5 (19.3)	− 0.06
Heart rate (bpm)	92.9 (25.5)	94.1 (25.5)	0.05	94.2 (25.4)	0.05	92.0 (24.79)	94.1 (25.5)	0.08	92.0 (24.8)	94.2 (25.4)	0.08
Glucose (mg/dL)	184.2 (76.0)	188.7 (109.1)	0.04	202.2 (108.3)	0.17^1^	183.8 (83.7)	188.7(109.1)	0.05	183.8 (83.7)	202.2 (108.3)	0.05
Sodium (mmol/L)	137.3 (4.6)	136.0 (5.4)	− 0.23^1^	136.2 (5.3)	− 0.21^1^	136.6 (4.9)	136.0 (5.4)	− 0.11	136.6 (4.9)	136.2 (5.3)	− 0.06
Potassium (mmol/L)	4.3 (0.6)	4.7 (0.9)	0.38^1^	4.6 (0.9)	0.36^1^	4.6 (0.8)	4.7 (0.9)	0.05	4.6 (0.8)	4.6 (0.9)	0.10
BUN (mg/dL)	24.7 (14.3)	34.3 (21.1)	0.46^1^	32.6 (20.0)	0.40^1^	33.2 (20.3)	34.3 (21.1)	0.05	33.2 (20.3)	32.6 (20.0)	0.10
Creatinine (mg/dL)	1.4 (1.0)	2.1 (1.9)	0.37^1^	1.9 (1.7)	0.31^1^	2.0 (1.6)	2.1 (1.9)	0.05	2.0 (1.6)	1.9 (1.7)	0.03

	N (%)	N (%)	ASD	N (%)	ASD	N (%)	N (%)	ASD	N (%)	N (%)	ASD
Female patients	291 (46.9)	310 (45.5)	− 0.03	512 (45.7)	− 0.03	182 (42.7)	310 (45.5)	0.06	301 (43.2)	512 (45.7)	0.05
Hypertension	462 (74.5)	436 (63.9)	− 0.22^1^	746 (66.6)	− 0.17^1^	309 (72.6)	436 (63.9)	− 0.18	512 (73.5)	746 (66.6)	− 0.15^1^
IHD	225 (36.3)	247 (36.2)	0.00	410 (36.6)	0.01	153 (36.0)	247 (36.2)	0.01	263 (37.7)	410 (36.6)	− 0.02
AF	182 (29.4)	177 (26.0)	− 0.08	254 (22.7)	− 0.16^1^	120 (28.1)	177 (26.0)	− 0.05	161 (23.1)	254 (22.7)	− 0.01
COPD	75 (12.1)	77 (11.3)	− 0.03	119 (10.6)	− 0.05	62 (14.6)	77 (11.3)	− 0.10	100 (14.3)	119 (10.6)	− 0.12
CKD	96 (15.5)	182 (26.7)	0.25^1^	278 (24.8)	0.22^1^	132 (31.0)	182 (26.7)	− 0.10	188 (27.0)	278 (24.8)	− 0.05
CVA	105 (16.9)	122 (17.9)	0.03	201 (18.0)	0.03	72 (17.0)	122 (17.9)	0.02	121 (17.3)	201 (18.0)	0.02
BNP or NT-proBNP ^2^ (pg/mL)	482 (77.7)	551 (80.8)	0.08	908 (81.1)	0.09	344 (80.8)	551 (80.8)	0.00	565 (81.1)	908 (81.1)	0.00
NYHA class III–IV	537 (86.6)	620 (90.9)	0.15^1^	1,006 (89.8)	0.11^1^	388 (91.1)	620 (90.9)	− 0.01	625 (89.6)	1006 (89.8)	0.01
LVEF (%)	36.4 (15.2)	36.7 (14.6)	0.02	36.6 (14.6)	0.02	36.2 (14.5)	36.7 (14.6)	0.03	36.2 (14.5)	36.6 (14.6)	− 0.01
Management at admission											
Diuretics, IV	542 (87.4)	616 (90.3)	0.10	1013 (90.5)	0.10	381 (89.4)	616 (90.3)	0.03	619 (88.8)	1,013 (90.5)	0.06
Inotropes, IV	163 (26.3)	417 (61.1)	0.72^1^	641 (57.2)	0.63^1^	223 (52.3)	417 (61.1)	0.18^1^	353 (50.6)	641 (57.2)	0.14^1^
Vasodilators, IV	274 (44.2)	427 (62.6)	0.38^1^	703 (62.8)	0.38^1^	226 (53.0)	427 (62.6)	0.20^1^	385 (55.2)	703 (62.8)	0.16^1^
Management at discharge											
ACEIs/ARBs	461 (74.4)	332 (48.7)	− 0.51^1^	621 (55.5)	− 0.38^1^	247 (57.9)	332 (48.7)	− 0.19^1^	430 (61.7)	621 (55.5)	− 0.13
Beta-blockers	338 (54.5)	281 (41.2)	− 0.27^1^	537 (48.0)	− 0.13^1^	190 (44.6)	281 (41.2)	− 0.07	340 (48.9)	537 (48.0)	− 0.02
MRAs	295 (47.6)	245 (35.9)	− 0.24^1^	443 (39.6)	− 0.16^1^	160 (37.5)	245 (35.9)	− 0.03	274 (39.3)	443 (39.6)	0.01
Warfarin	172 (27.7)	183 (26.8)	− 0.02	264 (23.6)	− 0.10	115 (26.9)	183 (26.8)	0.00	175 (25.1)	264 (23.6)	− 0.04

*IPTW* inverse probability treatment weight, *OHA* oral hypoglycemic agents, *SD* standard deviation, *ASD* absolute standard difference, *BMI* body mass index, *SBP* systolic blood pressure, *DBP* diastolic blood pressure, *BUN* blood urine nitrogen, *IHD* ischemic heart disease, *AF* atrial fibrillation, *COPD* chronic lung obstructive disease, *CKD* chronic kidney disease, *CVA* cerebrovascular attack, *BNP* B-type natriuretic peptide, *NT-proBNP* N-terminal pro b-type natriuretic peptide, *NYHA* new York heart association, *LVEF* left ventricular ejection fraction, *ACEI* angiotensin-converting enzyme inhibitor, *ARB* angiotensin receptor blocker, *MRA* mineralocorticoid receptor antagonist

^1^P < 0.05

^2^BNP ≥ 500 pg/mL or NT-proBNP ≥ 1000 pg/mL

When comparing patients in the combination insulin and OHAs group with those in the OHA-only group, patients receiving insulin were significantly more likely to be younger and have a lower BMI. They were also more likely to have chronic kidney disease and a more severe NYHA class, but less likely to have hypertension and atrial fibrillation. Patients receiving insulin had higher rates of inotrope and vasodilator use during the index admission. The prescription rates for ACEIs/ARBs, BBs, and MRAs were significantly lower in the combination of insulin and OHAs group (Table 1).

### Mortality of patients with diabetes mellitus and heart failure based on insulin therapy

All-cause mortality rate per 10 person-years of HF patients with DM were 1.45 (95% CI 1.29–1.61), 2.18 (95% CI 1.98–2.39), and 1.98 (95% CI 1.84–2.13) in the OHA alone, insulin alone, and combination of insulin and OHAs group, respectively (Additional file 1: Table S3).

Both the insulin-only and all-insulin groups showed significantly higher mortality than the OHA-only group (HR 1.27; 95% CI 1.09–1.49 for the insulin-only group; HR 1.21; 95% CI 1.07–1.36 for all-insulin group, respectively). After the double adjustment for the imbalanced covariates, both the insulin-only and all-insulin groups were associated with significantly higher mortality (HR 1.41; 95% CI 1.21–1.66 for the insulin-only group; HR 1.29; 95% CI 1.14–1.46 for the all-insulin group) than the OHA-only group (Table 2).

**Table 2:** Overall mortality according to diabetes therapy in the inverse probability of treatment weighted pseudo-cohort within the KorAHF registry

Original cohort				Weighted^a^ pseudo-cohort			
	Person-years	Death (N)	HR (95 % CI)^b^	Person-years	Death (N)	HR (95 % CI)^b^	HR (95% CI)^c^
OHA-only	2277	329	1.00	2277	244	1.00	1.00
Insulin-only	1983	432	1.45 (1.26–1.67)	1983	432	1.27 (1.09–1.49)	1.41 (1.21–1.66)
OHA-only	2277	329	1.00	2277	395	1.00	1.00
All insulin	3478	689	1.33 (1.17–1.52)	3478	689	1.21 (1.07–1.36)	1.29 (1.14–1.46)

*N* number, *HR* hazard ratio, *CI* confidence interval, *OHA* oral hypoglycemic agents

^a^Inverse probability of treatment (IPT)-weighted

^b^Crude HR (95% CI)

^c^Additionally adjusted for age, vasodilators management at admission and ACEIs/ARBs management at dischare in the first pseudo-cohort with 426 OHA group and 682 insulin only group; adjusted for age, hypertension and inotropes and vasodilators management at admission

**Fig. 1:**
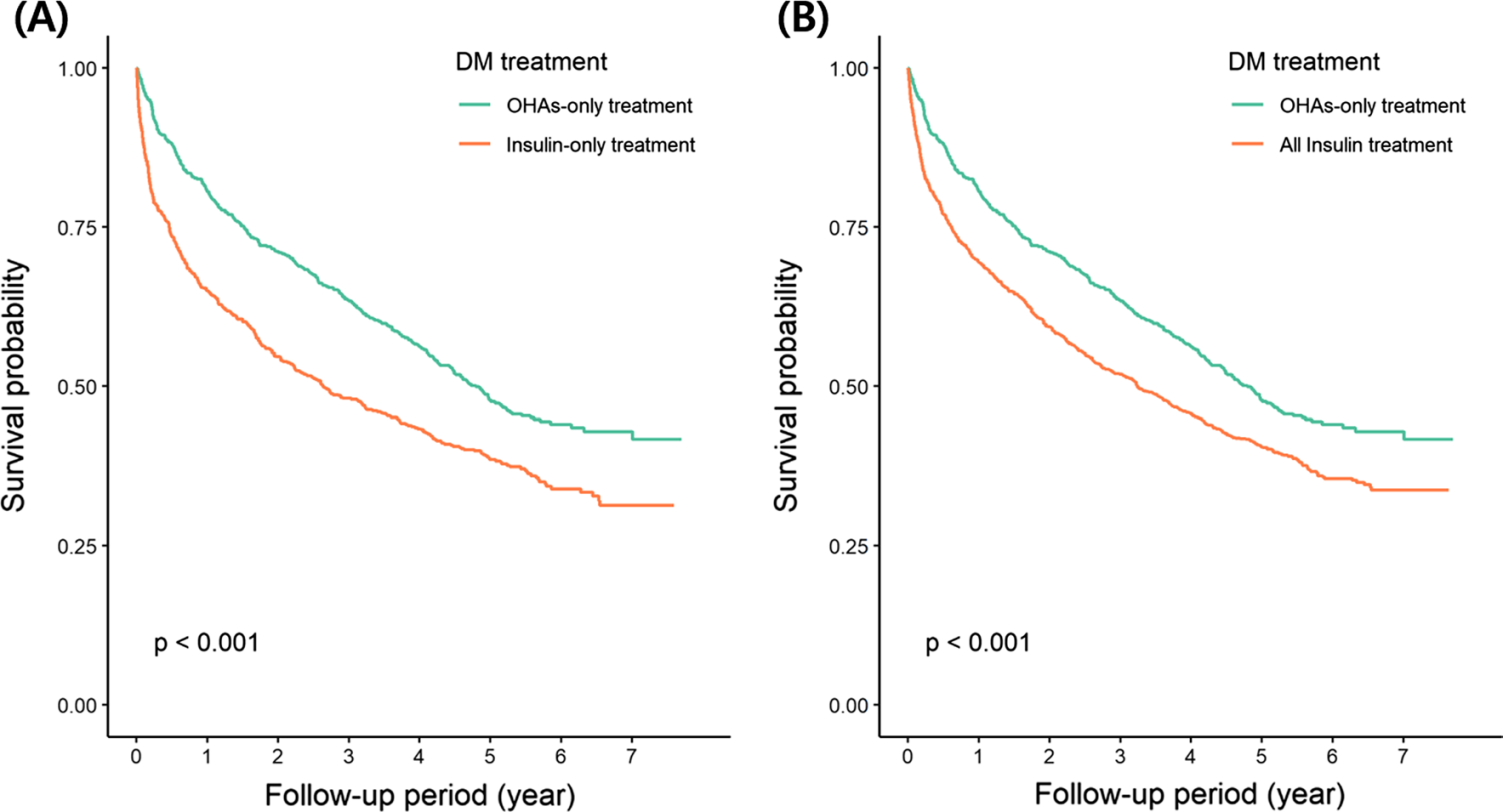
Kaplan–Meier survival curves for all-cause mortality according to the treatment type of diabetes mellitus after inverse probability treatment weighting. **A** Insulin-only group versus oral hypoglycemic agent (OHA)-only group. **B** All-insulin group versus OHA-only group

Kaplan–Meier survival analyses with IPTW demonstrate significantly higher mortality in both the insulin-only and all-insulin groups than in the OHA-only group. (Fig. 1).

### Impact of insulin therapy on the subgroups stratified according to age, sex, etiology of heart failure, left ventricular ejection fraction, and glycated hemoglobin level

**Fig. 2:**
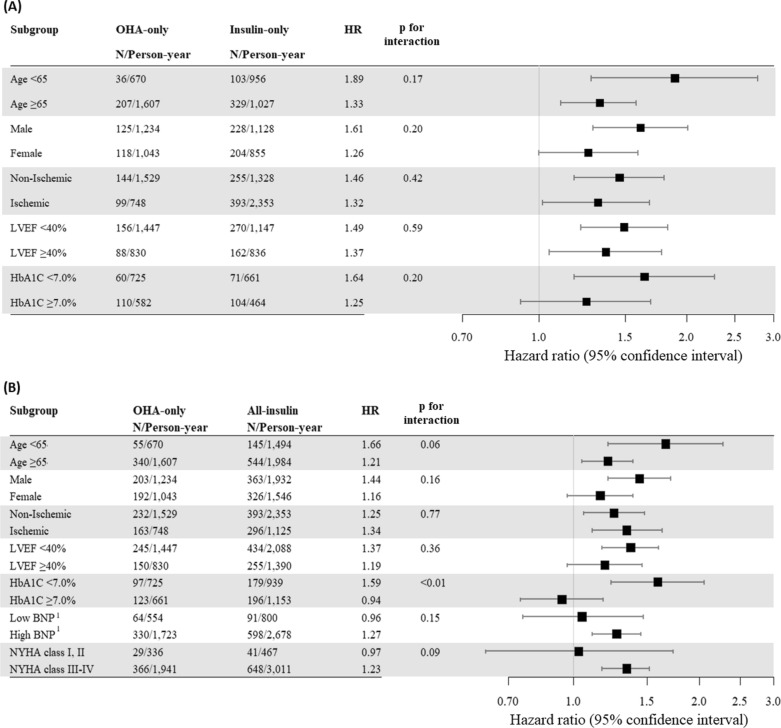
Subgroup analysis on the association between diabetes mellitus treatment type and overall mortality. Subgroup analyses on the association between diabetes mellitus treatment type and overall mortality according to age, sex, etiology of heart failure, left ventricular ejection fraction, glycosylated hemoglobin level, natriuretic peptide level, and NYHA class after double adjustment of weighted Cox proportional hazard regression model. **A** Insulin-only group versus oral hypoglycemic agent (OHA)-only group (reference). **B** All-insulin group versus OHA-only group. Low BNP and high BNP were defined as BNP < 500 pg/mL and NT-proBNP < 1000 pg/mL, and BNP ≥ 500 pg/mL or NT-proBNP ≥ 1000 pg/mL, respectively

The impact of insulin therapy on the subgroups classified according to age, sex, etiology of HF, LVEF, HbA1c level, natriuretic peptide level, and NYHA class in the setting of double adjustment is shown in Fig. 2. Insulin therapy was associated with a higher risk of mortality in both subgroups of patients aged < 65 years and ≥ 65 years than OHAs (Additional file 1: Table S4). Male sex was associated with significantly higher mortality in the insulin-only and all-insulin groups than in the OHA-only group. However, the higher mortality in the all-insulin group compared to that in the OHA-only group was not statistically significant among the female population (Additional file 1: Table S5). Insulin therapy was also associated with higher mortality than OHAs, regardless of HF etiology (ischemic or non-ischemic). (Additional file 1: Table S6).

Those in the insulin-only and all-insulin groups showed higher mortality than those in the OHA-only group among HF patients with reduced EF (LVEF < 40 %) (HR 1.49; 95% CI 1.22–1.83 for insulin-only and HR 1.37; 95% CI 1.17–1.60 for all-insulin, respectively). For patients with preserved EF (LVEF ≥ 40 %), although significant association between combination of insulin and OHA therapy and mortality was not found (HR 1.19; 95% CI 0.97–1.46), increased mortality was observed in the insulin-only group (HR 1.37; 95% CI 1.05–1.78) compared to that in the OHA-only group. (Additional file 1: Table S7).

The impact of insulin therapy on mortality was evaluated in the population stratified according to HbA1c levels with a cutoff of 7.0 %. In the patient subgroup with good glycemic control (HbA1c < 7.0 %), the insulin-only group (HR 1.64; 95% CI 1.18–2.28) and all-insulin group (HR 1.59; 95% CI 1.23–2.05) were associated with increased mortality compared with the OHA-only group. However, for patients with poor glycemic control (HbA1c ≥ 7.0 %), neither the insulin-only group nor the all-insulin group was associated with increased mortality compared with the OHA-only group (Additional file 1: Table S8). The impact of insulin therapy (all-insulin group) on mortality was significantly different according to HbA1c level (*p*-interaction < 0.01), indicating that the increased risk associated with insulin therapy was prominent in the subgroup of patients with low HbA1c (< 7.0 %) (Fig. 2B). Patients with good glycemic control with combined insulin and OHA therapy showed a similar mortality risk to that in patients with poor glycemic control (HbA1c ≥ 7.0 %) with OHA therapy alone (Table 3).

**Table 3:** Overall mortality according to diabetes therapy in the inverse probability of treatment weighted pseudo-cohort within the KorAHF registry

	Original cohort			Weighted^a^ pseudo-cohort			
	Person-years	Death N	HR (95% CI)^b^	Person-years	Death N	HR (95% CI)^b^	HR (95% CI)^c^
HbA1c < 7.0%							
OHA-only	725	85	1.00	725	60	1.00	1.00
Insulin-only	582	110	1.55 (1.16–2.05)	582	110	1.52 (1.11–2.09)	1.64 (1.18–2.28)
HbA1c ≥ 7.0%							
OHA-only	661	102	1.30 (0.98–1.74)	661	71	1.58 (1.12–2.23)	1.69 (1.18–2.42)
Insulin-only	464	104	1.84 (1.38–2.42)	464	104	1.83 (1.33–2.52)	2.25 (1.61–3.13)
* p*-interaction			0.66			0.22	0.20
HbA1c < 7.0%							
Only oral	725	85	1.00	725	97	1.00	1.00
All insulin	939	179	1.56 (1.20–2.02)	939	179	1.50 (1.17–1.92)	1.59 (1.23–2.05)
HbA1c ≥ 7.0%							
Only oral	661	102	1.30 (0.98–1.74)	661	123	1.52 (1.17–1.99)	1.58 (1.20–2.09)
All insulin	1153	196	1.42 (1.10–1.83)	1153	196	1.38 (1.08–1.76)	1.57 (1.22–2.02)
* p*-interaction			0.05			0.85	< 0.01

*N* number, *HR* hazard ratio, *CI* confidence interval, *OHA* oral hypoglycemic agents

^a^Inverse probability treatment (IPT)-weighted

^b^Crude HR (95% CI)

^c^Additionally adjusted for age, vasodilators management at admission and ACEIs/ARBs management at dischare in the first pseudo-cohort with 426 OHA group and 682 insulin only group; adjusted for age, hypertension and inotropes and vasodilators management at admission

 Lastly, the difference in the prognosis between the patients with and without insulin was tested according to the severity of heart failure classified using the NYHA class and natriuretic peptide level (Additional file 1: Table S9). The analysis was performed only between the group with OHA and all-insulin because of the small number of patients treated with insulin only and having mild symptoms (NYHA I–II) or low natriuretic peptide levels. In patients with NYHA class I–II, the mortality of all-insulin group did not differ from that with OHA (HR 1.03; 95% CI 0.62–1.73). However higher mortality related to insulin therapy was observed in patients NYHA class III–IV (HR 1.34; 95% CI 1.17–1.52). A similar trend was observed in the groups divided based on the natriuretic peptide level. In patients with high natriuretic peptide levels, insulin therapy (all-insulin group) was associated with worse outcomes (HR 1.27; 95% CI 1.11–1.45), whereas the all-insulin group did not present worse outcomes compared to those with OHA (HR 1.05; 95% CI 0.76–1.47) in those with low natriuretic peptide levels. Overall, the results may imply that the negative prognostic impact of insulin treatment tends to be prominent in patients with more severely presented heart failure.

Sulfonylurea and metformin were the most frequently used classes of OHAs in this study. There was no significant difference in mortality between the groups using sulfonylurea, metformin, or even in the case of the combination of both OHAs and those treated with insulin (Additional file 1: Table S10).

## Discussion

This study found that insulin therapy was associated with increased mortality in HF patients with DM, and this was consistent regardless of LVEF and HF etiology (ischemic or non-ischemic). This increase in mortality was predominantly observed in patients with good glycemic control (HbA1c < 7.0 %) or severe degrees of HF assessed using the NYHA class and natriuretic peptide levels.

The current evidence regarding the effect of insulin therapy on the prognosis of HF patients with DM is based on observational studies. Lawson et al. investigated the effect of DM on HF patients using the UK Clinical Practice Research Datalink, which covers 10% of the UK population [12]. HF patients treated with OHAs or insulin had an increased risk of all-cause hospitalization compared to HF patients without DM. Although they did not directly compare the DM treatment method, insulin therapy showed a greater numerical risk for hospitalization. Cooper et al. also evaluated the association between insulin therapy and prognosis in 35,603 patients with newly diagnosed DM from the Medicare claim data [14]. They found that patients with HF who were prescribed insulin had a higher incidence of death and hospitalization than those treated with OHAs. Moreover, DM independently predicted morbidity and mortality in chronic HF patients from the Candesartan in Heart Failure: Assessment of Reduction in Mortality and Morbidity program [18]. Patients with DM treated with and without insulin had higher cardiovascular mortality and HF hospitalization rates. A recent meta-analysis by Cosmi et al., which included the Valsartan Heart Failure Trial (Val-Heft), Controlled Rosuvastatin Multinational Trial in HF (CORONA), Gruppo Italiano per lo Studio della Sopravvivenza nell’Insufficienza Cardiaca-Heart Failure (GISSI-HF), and Aliskiren Trial to Minimize Outcomes in Patients with Heart Failure (ATMOSPHERE), showed that the rate of all-cause mortality and HF hospitalization was higher in patients with DM than in those without, and the highest was for the patient group prescribed insulin [18–22]. In contrast to these findings, Masoudi et al. found no clear association between insulin use and the prognosis of HF patients, after having enrolled 16,417 HF patients with DM from the Medicare data [23], and found that insulin therapy was not associated with the 1-year overall mortality.

Although there is some controversy regarding the impact of insulin therapy on HF patients, numerous studies have described the adverse effects, other than mortality, in these patients. Skott et al. investigated the effects of insulin on kidney function and sodium excretion. They found that the increased insulin levels, induced by a 120-min infusion, showed a marked increase in sodium reabsorption, even within the physiological range [24]. Insulin-induced sodium and water retention are likely to exacerbate cardiac congestion, leading to acute HF decompensation and increasing the need for loop diuretics [24, 25]. These adverse effects can be more critical in those with severe degrees of HF. The results from the present study demonstrated that the adverse effect of insulin was prominent in patients with severe degrees of HF, which can be understood in the same context. The negative prognostic impact of insulin showed a similar trend in both insulin-only and all-insulin groups, indicating that the effect may be rather due to insulin itself, not the combination of insulin and other OHA.

In addition, insulin use increases the risk of hypoglycemia, which can stimulate the autonomic nervous system as a counter-regulatory response. Laitinen et al. demonstrated that hyperinsulinemic hypoglycemia could cause a 12-fold increase in plasma epinephrine levels, which can induce a substantial increase in myocardial contractility, myocardial oxygen demand, heart rate, and cardiac output. These hemodynamic changes, coupled with a shortage of glucose supply, can be deleterious for patients with HF. The study conducted by Bendenis et al. showed an increase in hypoglycemia-induced cardiovascular events and an increased risk of coronary heart disease in patients with recent hypoglycemic events compared with those in patients without such events. The study also revealed that hypoglycemia is associated with systemic inflammation, inferred by the increase in inflammatory biomarkers [26]. In our study, the adverse effect of increased mortality associated with insulin therapy was predominantly observed in patients with low HbA1c levels (< 7.0 %). Although data were not collected on the hypoglycemic events in our study, insulin-induced hypoglycemic events followed by catecholamine surges and systemic inflammation could be a plausible explanation for the increased mortality of patients with low HbA1c levels receiving insulin therapy. Therefore, the optimal timing for initiating insulin therapy, as well as the target level for glucose control in HF patients with DM might be different from patients with DM alone and warrants further investigation in future studies.

In the meta-analysis by Cosmi et al. [22], the clinical trials Val-Heft, CORONA, and ATMOSPHERE included HF patients with reduced LVEF. They consistently showed an association between insulin use and poor prognosis in HF patients. The HRs for all-cause mortality were 1.23 (95% CI 0.98–1.55), 1.27 (95% CI 1.05–1.53), and 1.23 (95% CI 1.09–1.45) for Val-Heft, CORONA, and ATMOSPHERE, respectively. The GISSI-HF trial, which included HF patients regardless of LVEF, still showed an increased risk of mortality with insulin compared to that without insulin therapy (HR, 1.29; 95% CI 1.10–1.51). The subgroup analysis of the Treatment of Preserved Cardiac Function Heart Failure with an Aldosterone Antagonist study, which only enrolled HF patients with preserved EF (LVEF  ≥ 45%), showed that insulin therapy was associated with a 40 % increase in all-cause and cardiovascular mortality compared with DM therapy without insulin [27]. Our study showed the adverse impact of increased mortality in HF patients receiving insulin therapy across LVEF ranges in a single study cohort [both preserved EF (LVEF  ≥ 40%) and reduced EF (LVEF < 40%)].

## Study limitations

This study has some limitations. First, due to its observational nature, there were differences in the clinical characteristics between the two groups. Although we initially tried to estimate the IPTW using a logistic regression model, it was difficult to balance the characteristics between the untreated group and the treated group by using this method. Therefore, we applied the generalized boosted model to estimate the IPTW, which can consider many pretreatment covariates and reflect the nonlinear and complex associations between pretreatment covariates and treatment. Although the Cox proportional hazard model with double adjustment for the imbalanced variables and IPTW was performed, unmeasured confounding may still exist. Well-designed clinical trials will be needed to elucidate the adverse effect of insulin therapy in patients with HF more clearly.

Second, patients taking sodium-glucose co-transporter-2 (SGLT-2) inhibitors were not included as they were not available in clinical practice in the Republic of Korea during the enrollment period (2011–2014) of the KorAHF. Recent studies have demonstrated the benefits of SGLT-2 inhibitors in HF patients [28], whereas most of the other OHAs showed neutral effects in these patients [29]. The inclusion of SGLT-2 inhibitors could have caused an analytic bias, widening the gap between insulin therapy and other hypoglycemic agents. Data regarding glucagon-like peptide 1 receptor agonist (GLP1RA) is not available either in the analysis. The reason is that the GLP1RA prescription rate was very low during the enrollment period (2011–2014) of the KorAHF registry because few drugs were available in Korea at that time and there were some limitations related to the healthcare system.

Third, only limited information on DM medication was available such as the usage and type of DM medication at the baseline. Although the adjustment of anti-diabetic treatment and patients’ adherence during the follow-up are also significant factors related to the prognosis, we could not consider them in the current analyses.

Lastly, the latency period between hypoglycemic therapy and death was not considered in this study, since there was no information on the duration of DM medication prior to enrollment and the number of subjects was not sufficient to exclude those who died immediately after the defined drug exposure time point (enrollment date).

## Conclusions

Insulin therapy in HF patients with DM is associated with a higher mortality risk than OHAs, regardless of the patients’ LVEF and HF etiology. The result of this paper suggested that insulin therapy was harmful, especially in patients with low HbA1c levels or more severe forms of heart failure; therefore, specific management strategies and blood sugar targets may be needed when using insulin in patients with HF.

## Supplementary Information

**Additional file 1: Figure S1.** Flow chart for study subject selection. **Table S1.** Composition of the oral hypoglycemic medication for the OHA-only group and both OHAs and insulin group. **Table S2.** Insulin treatment in KorAHF registry. **Table S3.** Mortality rates according to hypoglycemic therapy among the patients with diabetes and heart failure. **Table S4.** Association between diabetes therapy and overall mortality according to age in the pseudo-cohort. **Table S5.** Association between diabetes therapy and overall mortality according to sex in the pseudo-cohort. **Table S6.** Association between diabetes therapy and overall mortality according to ischemic etiology in the pseudo-cohort. **Table S7.** Association between diabetes therapy and overall mortality according to left ventricular ejection fraction in the pseudo-cohort. **Table S8.** Association between diabetes therapy and overall mortality according to glycated hemoglobin levels in the pseudo-cohort. **Table S9.** Association between diabetes therapy and overall mortality according to severity of heart failure in the pseudo-cohort. **Table S10.** Overall mortality according to diabetes therapy in stratified population by diabetes mellitus medication in the weighted pseudo-cohort.

## Data Availability

The data of this study may be available on reasonable request to the Korean Acute Heart Failure.
