# Supplementary material for: Impact of insulin therapy on the mortality of acute heart failure patients with diabetes mellitus
Source: Cardiovasc Diabetol. 2021 Sep 8;20:180. doi: 10.1186/s12933-021-01370-y (PMC8424885; doi:10.1186/s12933-021-01370-y)
Supplement: Supplementary file 1 — Additional file 1: Figure S1. Flow chart for study subject selection. Table S1. Composition of the oral hypoglycemic medication for the OHA-only group and both OHAs and insulin group. Table S2. Insulin treatment in KorAHF registry. Table S3. Mortality rates according to hypoglycemic therapy among the patients with diabetes and heart failure. Table S4. Association between diabetes therapy and overall mortality according to age in the pseudo-cohort. Table S5. Association between diabetes therapy and overall mortality according to sex in the pseudo-cohort. Table S6. Association between diabetes therapy and overall mortality according to ischemic etiology in the pseudo-cohort. Table S7. Association between diabetes therapy and overall mortality according to left ventricular ejection fraction in the pseudo-cohort. Table S8. Association between diabetes therapy and overall mortality according to glycated hemoglobin levels in the pseudo-cohort. Table S9. Association between diabetes therapy and overall mortality according to severity of heart failure in the pseudo-cohort. Table S10. Overall mortality according to diabetes therapy in stratified population by diabetes mellitus medication in the weighted pseudo-cohort. [file 12933_2021_1370_MOESM1_ESM.docx]

**Additional file 1**

**Figure S1. Flow chart for study subject selection**


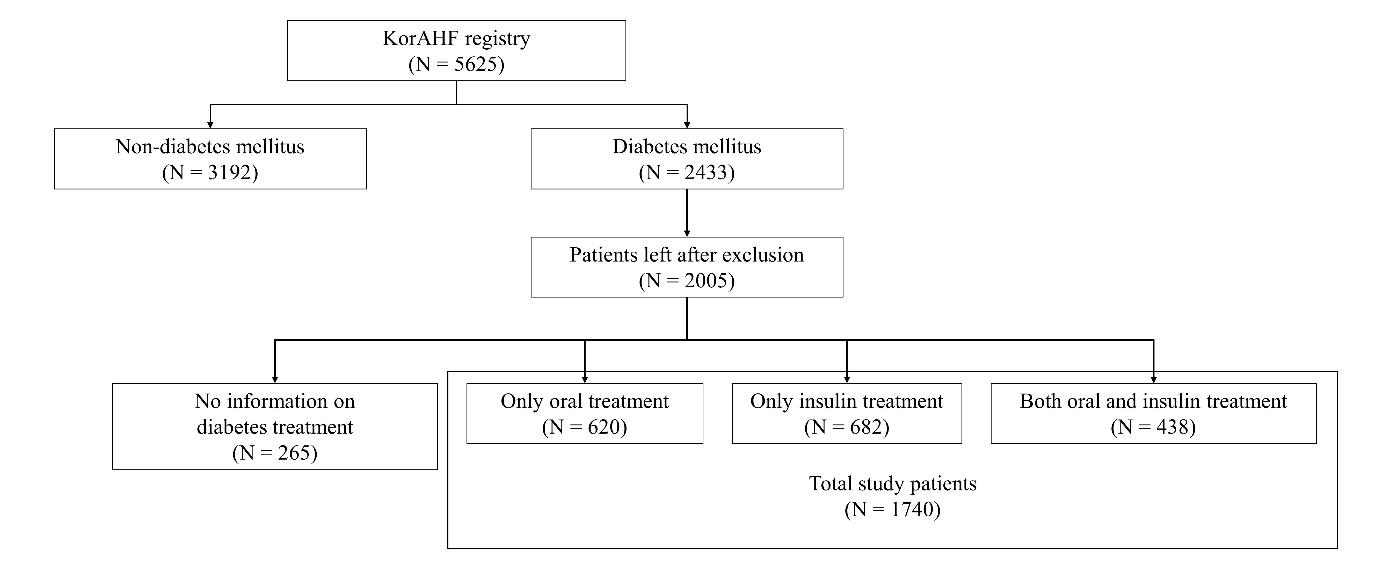


| **Table S1. Composition of the oral hypoglycemic medication for the OHA-only group and both OHAs and insulin group** | | |
| --- | --- | --- |
| **Type of medication** | **OHA-only group  (N = 620)** | **Both OHAs and insulin group (N = 438)** |
|  | N (%) | N (%) |
| Sulfonylurea | 363 (58.6) | 275 (62.8) |
| Metformin | 368 (59.4) | 246 (56.2) |
| Thiazolidinedione | 22 (3.6) | 13 (3.0) |
| Acarbose | 79 (12.7) | 63 (14.4) |
| Dipeptidyl peptidase 4 inhibitor | 117 (18.9) | 87 (19.9) |

Abbreviation: OHA, Oral hypoglycemic agents; N, Number

**Table S2. Insulin treatment in KorAHF registry**

| **Type of insulin** | **Prescription (%)** | **Dose (unit) Median (IQR)** |
| --- | --- | --- |
| **Intermediate-acting insulin** | 38.9 |  |
| Neutral Protamine Hagedorn (NPH) insulin | 38.9 | 10 (6-20) |
| **Long acting insulin** | 21.3 |  |
| Insulin glargine | 19.9 | 17 (12-24) |
| Insulin detemir | 1.4 | 16 (12-22) |
| **Short acting insulin** | 57.4 |  |
| Insulin aspart | 0.4 | 18 |
| Insulin glulisine | 10.9 | 12 (7-21) |
| Insulin lispro | 4.1 | 6 (4-15) |
| Regular insulin | 42.0 | 15 (10-40) |
| **Premixed insulin** | 13.9 |  |
| Insulin aspart protamine + insulin aspart injectable suspension, 70/30 | 9.4 | 35 (26-40) |
| Insulin lispro protamine and insulin lispro injectable suspension, 75/25 | 3.1 | 44 (22-56) |
| Insulin lispro protamine and insulin lispro injectable suspension, 50/50 | 1.2 | 42 (26-48) |
| Neutral Protamine Hagedorn (NPH) insulin / regular insulin 70/30 | 0.2 | 38 |

Abbreviation: IQR, Interquartile range

| **Table S3. Mortality rates according to hypoglycemic therapy among the patients with diabetes and heart failure** | | | | |
| --- | --- | --- | --- | --- |
|  | Untreated  (N **=** 265) | OHA-Only group  (N **=** 620) | Insulin-only group  (N **=** 682) | All insulin group  (N **=** 1120) |
| Person-years | 859 | 2277 | 1983 | 3478 |
| N of deaths | 162 | 329 | 432 | 689 |
| All-cause mortality  per 10 person-years (95% CI) | 1.89 (1.61–2.20) | 1.45 (1.29–1.61) | 2.18 (1.98–2.39) | 1.98 (1.84–2.13) |

Abbreviation: N, Number; CI, Confidence interval

**Table S4. Association between diabetes therapy and overall mortality according to age in the pseudo-cohort**

|  | **Original cohort** | | | **Weighted^1^ pseudo-cohort** | | | |
| --- | --- | --- | --- | --- | --- | --- | --- |
|  | Person-years | Death  N | HR (95% CI)^2^ | Person-years | Death  N | HR (95% CI)^2^ | HR (95% CI)^3^ |
| **Age <65-year** | | | | | | | |
| OHA-only | 670 | 41 | 1.00 | 670 | 36 | 1.00 | 1.00 |
| Insulin-only | 956 | 103 | 1.70 (1.18–2.44) | 956 | 103 | 1.63 (1.12–2.39) | 1.89 (1.28–2.79) |
| OHA-only | 670 | 41 | 1.00 | 670 | 55 | 1.00 | 1.00 |
| All insulin | 1494 | 145 | 1.55 (1.10–2.19) | 1494 | 145 | 1.59 (1.16–2.16) | 1.66 (1.21–2.28) |
| **Age ≥65-year** | | | | | | | |
| OHA-only | 1607 | 288 | 1.00 | 1607 | 207 | 1.00 | 1.00 |
| Insulin-only | 1027 | 329 | 1.68 (1.43-1.97) | 1027 | 329 | 1.32 (1.11-1.57) | 1.33 (1.11–1.58) |
| OHA-only | 1607 | 288 | 1.00 | 1607 | 340 | 1.00 | 1.00 |
| All insulin | 1984 | 544 | 1.47 (1.27-1.69) | 1984 | 544 | 1.20 (1.05-1.38)) | 1.21 (1.05–1.39) |

Abbreviation: N, Number; HR, Hazard ratio; OHA, Oral hypoglycemic agents

1. Inverse probability treatment-weighted

2. Crude HR (95% CI)

3. Additionally adjusted for age, vasodilators management at admission and ACEIs/ARBs management at discharge in the first pseudo-cohort with 426 OHA group and 682 insulin only group; adjusted for age, hypertension and inotropes and vasodilators management at admission

**Table S5. Association between diabetes therapy and overall mortality according to sex in the pseudo-cohort**

|  | **Original cohort** | | | **Weighted^1^ pseudo-cohort** | | | |
| --- | --- | --- | --- | --- | --- | --- | --- |
|  | Person-years | Death  N | HR (95% CI)^2^ | Person-years | Death  N | HR (95% CI)^2^ | HR (95% CI)^3^ |
| **Male** | | | | | | | |
| OHA-only | 1234 | 168 | 1.00 | 1234 | 125 | 1.00 | 1.00 |
| Insulin-only | 1128 | 228 | 1.45 (1.18–1.77) | 1128 | 228 | 1.41 (1.14–1.76) | 1.61 (1.29–2.01) |
| OHA-only | 1234 | 168 | 1.00 | 1234 | 203 | 1.00 | 1.00 |
| All insulin | 1932 | 363 | 1.35 (1.13–1.62) | 1932 | 363 | 1.35 (1.14–1.60) | 1.44 (1.21–1.71) |
| **Female** | | | | | | | |
| OHA-only | 1043 | 161 | 1.00 | 1043 | 118 | 1.00 | 1.00 |
| Insulin-only | 855 | 204 | 1.47 (1.19–1.80) | 855 | 204 | 1.11 (0.88–1.39) | 1.26 (1.00–1.59) |
| OHA-only | 1043 | 161 | 1.00 | 1043 | 192 | 1.00 | 1.00 |
| All insulin | 1546 | 326 | 1.32 (1.09–1.59) | 1546 | 326 | 1.05 (0.88–1.25) | 1.16 (0.97–1.39) |

Abbreviation: N, Number; HR, Hazard ratio; OHA, Oral hypoglycemic agents

1. Inverse probability treatment-weighted

2. Crude HR (95% CI)

3. Additionally adjusted for age, vasodilators management at admission and ACEIs/ARBs management at discharge in the first pseudo-cohort with 426 OHA group and 682 insulin only group; adjusted for age, hypertension and inotropes and vasodilators management at admission

**Table S6. Association between diabetes therapy and overall mortality according to ischemic etiology in the pseudo-cohort**

|  | **Original cohort** | | | **Weighted^1^ pseudo-cohort** | | | |
| --- | --- | --- | --- | --- | --- | --- | --- |
|  | Person-years | Death  N | HR (95% CI)^2^ | Person-years | Death  N | HR (95% CI)^2^ | HR (95% CI)^3^ |
| **Non-ischemic etiology** | | | | | | | |
| OHA-only | 1529 | 192 | 1.00 | 1529 | 144 | 1.00 | 1.00 |
| Insulin-only | 1328 | 255 | 1.46 (1.21–1.76) | 1328 | 255 | 1.25 (1.02–1.53) | 1.46 (1.18–1.80) |
| OHA-only | 1529 | 192 | 1.00 | 1529 | 232 | 1.00 | 1.00 |
| All insulin | 2353 | 393 | 1.29 (1.09–1.53) | 2353 | 393 | 1.12 (0.95–1.31) | 1.25 (1.06–1.48) |
| **Ischemic etiology** | | | | | | | |
| OHA-only | 748 | 137 | 1.00 | 748 | 99 | 1.00 | 1.00 |
| Insulin-only | 655 | 177 | 1.44 (1.15–1.80) | 655 | 177 | 1.31 (1.02–1.67) | 1.32 (1.02–1.68) |
| OHA-only | 748 | 137 | 1.00 | 748 | 163 | 1.00 | 1.00 |
| All insulin | 1125 | 296 | 1.41 (1.15–1.72) | 1125 | 296 | 1.38 (1.14–1.67) | 1.34 (1.11–1.63) |

Abbreviation: N, Number; HR, Hazard ratio; OHA, Oral hypoglycemic agents

1. Inverse probability treatment-weighted

2. Crude HR (95% CI)

3. Additionally adjusted for age, vasodilators management at admission and ACEIs/ARBs management at discharge in the first pseudo-cohort with 426 OHA group and 682 insulin only group; adjusted for age, hypertension and inotropes and vasodilators management at admission

**Table S7. Association between diabetes therapy and overall mortality according to left ventricular ejection fraction in the pseudo-cohort**

|  | **Original cohort** | | | **Weighted^1^ pseudo-cohort** | | | |
| --- | --- | --- | --- | --- | --- | --- | --- |
|  | Person-years | Death  N | HR (95% CI)^2^ | Person-years | Death  N | HR (95% CI)^2^ | HR (95% CI)^3^ |
| **LVEF <40%** | | | | | | | |
| OHA-only | 1447 | 202 | 1.00 | 1447 | 156 | 1.00 | 1.00 |
| Insulin-only | 1147 | 270 | 1.58 (1.31–1.89) | 1147 | 270 | 1.34 | 1.49 (1.22–1.83) |
| OHA-only | 1447 | 202 | 1.00 | 1447 | 245 | 1.00 | 1.00 |
| All insulin | 2088 | 434 | 1.42 (1.20–1.68) | 2088 | 434 | 1.26 (1.08–1.47) | 1.37 (1.17–1.60) |
| **LVEF ≥40%** | | | | | | | |
| OHA-only | 830 | 127 | 1.00 | 830 | 88 | 1.00 | 1.00 |
| Insulin-only | 836 | 162 | 1.25 (0.99–1.58) | 836 | 162 | 1.17 (0.90–1.51) | 1.37 (1.05–1.78) |
| OHA-only | 830 | 127 | 1.00 | 830 | 150 | 1.00 | 1.00 |
| All insulin | 1390 | 255 | 1.19 (0.96–1.47) | 1390 | 255 | 1.11 (0.91–1.36) | 1.19 (0.97–1.46) |

Abbreviation: N, Number; HR, Hazard ratio; OHA, Oral hypoglycemic agents

1. Inverse probability treatment-weighted

2. Crude HR (95% CI)

3. Additionally adjusted for age, vasodilators management at admission and ACEIs/ARBs management at discharge in the first pseudo-cohort with 426 OHA group and 682 insulin only group; adjusted for age, hypertension and inotropes and vasodilators management at admission

**Table S8. Association between diabetes therapy and overall mortality according to glycated hemoglobin levels in the pseudo-cohort**

|  | **Original cohort** | | | **Weighted^1^ pseudo-cohort** | | | |
| --- | --- | --- | --- | --- | --- | --- | --- |
|  | Person-years | Death  N | HR (95% CI)^2^ | Person-years | Death  N | HR (95% CI)^2^ | HR (95% CI)^3^ |
| **HbA1c <7.0%** | | | | | | | |
| OHA-only | 725 | 85 | 1.00 | 725 | 116 | 1.00 | 1.00 |
| Insulin-only | 582 | 110 | 1.55 (1.16–2.05) | 582 | 74 | 1.52 (1.11–2.09) | 1.64 (1.18–2.28) |
| OHA-only | 725 | 85 | 1.00 | 725 | 74 | 1.00 | 1.00 |
| All insulin | 939 | 179 | 1.56 (1.20–2.02) | 939 | 46 | 1.50 (1.17–1.92) | 1.59 (1.23–2.05) |
| **HbA1c ≥7.0%** | | | | | | | |
| OHA-only | 661 | 102 | 1.00 | 661 | 71 | 1.00 | 1.00 |
| Insulin-only | 464 | 104 | 1.43 (1.09–1.88) | 464 | 104 | 1.18 (0.87–1.60) | 1.25 (0.92–1.69) |
| OHA-only | 661 | 102 | 1.00 | 661 | 123 | 1.00 | 1.00 |
| All insulin | 1153 | 196 | 1.11 (0.87–1.41) | 1153 | 196 | 0.92 (0.73–1.16) | 0.94 (0.75–1.18) |

Abbreviation: N, Number; HR, Hazard ratio; OHA, Oral hypoglycemic agents

1. Inverse probability treatment-weighted

2. Crude HR (95% CI)

3. Additionally adjusted for age, vasodilators management at admission and ACEIs/ARBs management at discharge in the first pseudo-cohort with 426 OHA group and 682 insulin only group; adjusted for age, hypertension and inotropes and vasodilators management at admission

**Table S9.** **Association between diabetes therapy and overall mortality according to severity of heart failure in the pseudo-cohort**

|  | **Original cohort** | | | **Weighted^1^ pseudo-cohort** | | | |
| --- | --- | --- | --- | --- | --- | --- | --- |
|  | Person-years | Death  N | HR (95% CI)^2^ | Person-years | Death  N | HR (95% CI)^2^ | HR (95% CI)^3^ |
| **NYHA class I-II** | | | | | | | |
| OHA-only | 336 | 38 | 1.00 | 336 | 29 | 1.00 | 1.00 |
| All insulin | 467 | 41 | 0.78 (0.50-1.21) | 467 | 41 | 0.87 (0.54-1.39) | 1.03 (0.62-1.73) |
| **NYHA class III-IV** | | | | | | | |
| OHA-only | 1,941 | 291 | 1.00 | 1,941 | 366 | 1.00 | 1.00 |
| All insulin | 3,011 | 648 | 1.39 (1.21-1.60) | 3,011 | 648 | 1.24 (1.09-1.41) | 1.34 (1.17-1.52) |
| **BNP < 500pg/mL and NT-proBNP <1000pg/mL** | | | | | | | |
| OHA-only | 554 | 61 | 1.00 | 554 | 64 | 1.00 | 1.00 |
| All insulin | 800 | 91 | 1.04 (0.75-1.43) | 800 | 91 | 0.90 (0.66-1.24) | 1.05 (0.76-1.47) |
| **BNP ≥ 500pg/mL or NT-proBNP ≥1000pg/mL** | | | | | | | |
| OHA-only | 1,723 | 268 | 1.00 | 1,723 | 330 | 1.00 | 1.00 |
| All insulin | 2,678 | 598 | 1.39 (1.20-1.61) | 2,678 | 598 | 1.28 (1.12-1.47) | 1.27 (1.11-1.45) |

Abbreviation: BNP, B-type natriuretic peptide; NT-proBNP, N-terminal pro-B-type natriuretic peptide; NYHA, New York Heart Association

1. Inverse probability treatment-weighted

2. Crude HR (95% CI)

3. Additionally adjusted for age, vasodilators management at admission and ACEIs/ARBs management at discharge in the first pseudo-cohort with 426 OHA group and 682 insulin only group; adjusted for age, hypertension and inotropes and vasodilators management at admission

**Table S10. Overall mortality according to diabetes therapy in stratified population by diabetes mellitus medication in the weighted pseudo-cohort**

|  | **Original cohort** | | | **Weighted^1^ pseudo-cohort** | | | |
| --- | --- | --- | --- | --- | --- | --- | --- |
|  | Person-years | Death  N | HR (95% CI)^2^ | Person-years | Death  N | HR (95% CI)^2^ | HR (95% CI)^3^ |
| **Sulfonylurea only** | | | | | | | |
| OHA-only | 559 | 82 | 1.00 | 559 | 116 | 1.00 | 1.00 |
| All insulin | 347 | 74 | 1.41 (1.03–1.93) | 347 | 74 | 1.32 (0.98–1.77) | 1.38 (0.75–2.53) |
| **Metformin only** | | | | | | | |
| OHA-only | 585 | 70 | 1.00 | 585 | 74 | 1.00 | 1.00 |
| All insulin | 296 | 46 | 1.27 (0.87-1.84) | 296 | 46 | 1.08 (0.75-1.55) | 1.30 (0.61-2.78) |
| **Sulfonylurea and Metformin** | | | | | | | |
| OHA-only | 423 | 57 | 1.00 | 423 | 64 | 1.00 | 1.00 |
| All insulin | 307 | 42 | 1.01 (0.67-1.50) | 307 | 42 | 0.92 (0.62-1.36) | 0.85 (0.35-2.09) |

Abbreviation: N, Number; HR, Hazard ratio; OHA, Oral hypoglycemic agents

1. Inverse probability treatment-weighted

2. Crude HR (95% CI)

3. Additionally adjusted for age, vasodilators management at admission and ACEIs/ARBs management at discharge in the first pseudo-cohort with 426 OHA group and 682 insulin only group; adjusted for age, hypertension and inotropes and vasodilators management at admission
